# Profile of coronavirus disease enlightened asthma as a protective factor against death: An epidemiology study from Brazil during the pandemic

**DOI:** 10.3389/fmed.2022.953084

**Published:** 2022-11-29

**Authors:** Nathalia Mariana Santos Sansone, Felipe Eduardo Valencise, Rafael Fumachi Bredariol, Andressa Oliveira Peixoto, Fernando Augusto Lima Marson

**Affiliations:** ^1^Laboratory of Cell and Molecular Tumor Biology and Bioactive Compounds, São Francisco University, São Paulo, Brazil; ^2^Laboratory of Human and Medical Genetics, São Francisco University, São Paulo, Brazil; ^3^Center for Pediatric Investigation, University of Campinas, São Paulo, Brazil

**Keywords:** asthma, Brazil, COVID-19, pandemic, protective factor, pulmonary disease, risk factor, severe acute respiratory syndrome coronavirus 2

## Abstract

**Introduction:**

The possibility that asthma is not a risk factor for the worst outcomes due to coronavirus disease (COVID-19) is encouraged. The increase in Th2 response dominance can downregulate the late phase of hyperinflammation, which is typically the hallmark of more severe respiratory viral infections, alongside lower angiotensin-converting enzyme receptors in patients with asthma due to chronic inflammation. Few studies associated asthma diagnosis and COVID-19 outcomes. In this context, we aimed to associate the asthma phenotype with the clinical signs, disease progression, and outcomes in patients with COVID-19.

**Methods:**

We performed an epidemiologic study using patients’ characteristics from OpenDataSUS to verify the severity of COVID-19 among Brazilian hospitalized patients with and without the asthma phenotype according to the need for intensive care units, intubation, and deaths. We also evaluated the demographic data (sex, age, place of residence, educational level, and race), the profile of clinical signs, and the comorbidities.

**Results:**

Asthma was present in 43,245/1,129,838 (3.8%) patients. Among the patients with asthma, 74.7% who required invasive ventilatory support evolved to death. In contrast, 78.0% of non-asthmatic patients who required invasive ventilatory support died (OR = 0.83; 95% CI = 0.79–0.88). Also, 20.0% of the patients with asthma that required non-invasive ventilatory support evolved to death, while 23.5% of non-asthmatic patients evolved to death (OR = 0.81; 95% CI = 0.79–0.84). Finally, only 11.2% of the patients with asthma who did not require any ventilatory support evolved to death, while 15.8% of non-asthmatic patients evolved to death (OR = 0.67; 95% CI = 0.62–0.72). In our multivariate analysis, one comorbidity and one clinical characteristic stood out as protective factors against death during the severe acute respiratory syndrome coronavirus 2 (SARS-CoV-2) infection. Patients with asthma were less prone to die than other patients (OR = 0.79; 95% CI = 0.73–0.85), just like puerperal patients (OR = 0.74; 95% CI = 0.56–0.97) compared to other patients.

**Conclusion:**

Asthma was a protective factor for death in hospitalized patients with COVID-19 in Brazil. Despite the study’s limitations on patients’ asthma phenotype information and corticosteroid usage, this study brings to light information regarding a prevalent condition that was considered a risk factor for death in COVID-19, being ultimately protective.

## Introduction

Asthma is a prevalent chronic disease, affecting up to 4.4% of the world’s population. According to the World Health Survey, the global prevalence rates were 4.3% doctor-diagnosed asthma, 4.5% clinical/treated asthma, and 8.6% wheezing in adults and varied by as much as 21-fold amongst the 70 countries (1, 2). Asthma prevalence varies among countries, ranging from 21% in Australia to less than 2% in China (3). In Brazil, asthma prevalence is about 10% of the population (4). Due to its recurrent nature, it’s common for patients with asthma to experience exacerbations that lead to hospitalizations; also, the mortality rate due to asthma varies from two to 4/100,000 (2–4).

Few studies associated asthma phenotype and the coronavirus disease (COVID-19) prevalence, disease progression, and outcomes in the literature (2, 4–6). Curiously, it is not clear the characterization of asthma as a risk factor for the worst severity among patients with COVID-19 yet (7, 8). The clinical signs of asthma and COVID-19 are similar, presenting mainly cough and dyspnea. Also, fever is expected during COVID-19 and asthma exacerbations (9, 10). Nonetheless, respiratory viruses can trigger asthma exacerbations, increasing the severity of the infectious condition (4).

In this matter, past Coronavirus and Influenza viruses were considered triggers (5). As for the novel coronavirus, severe acute respiratory syndrome coronavirus 2 (SARS-CoV-2), it is still not clear whether asthma plays a role in a premorbid condition that represents a higher risk for COVID-19 prevalence and severity or plays a protective factor against the development of the disease (clinical signs) and its severity (3, 5). Several studies demonstrated a low prevalence of asthma among patients with COVID-19. For example, a Chinese study with 140 hospitalized patients did not mention asthma as a comorbidity factor for COVID-19 (11). In addition, in a study in Prato, Italy, carried out with 275 individuals, only three patients had asthma, and only one of them needed treatment in intensive care units (ICUs) (12). Furthermore, a study evaluated 2,500 patients in the city’s asthma-centered health department, and none required hospitalization due to SARS-CoV-2 infection (13). In this scenario, asthma was not considered a risk factor for severe COVID-19 or death due to the disease.

The immune response in viral infections is characterized by an activation of innate immunity with the production of interferons alpha (IFN-α), beta (IFN-β), and gamma (IFN-γ), which are responsible for containing the viral spread (14). Next, plasma and dendritic cells are responsible for the increased production of peripheral IFN-α (15). Interestingly, atopic patients have a low production of interferons, which constitutes an inadequate antiviral defense (15). Interferons act as negative regulators of the Th2 response, and there is a link between immunoglobulin E (IgE) and IFN-α, as IgE downregulates Toll-like (TLR)-9 receptors, reducing the interferon production by the dendritic cells (14, 16). The antiviral response is impaired due to the physiological pathway in atopic patients with asthma, who are more susceptible to viral infections, and these are predominant triggers of exacerbations (14–16).

**FIGURE 1:**
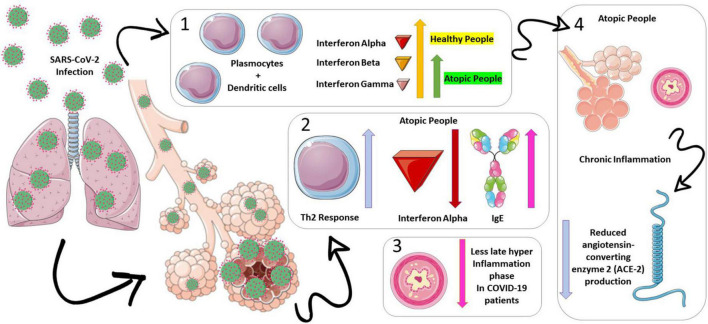
Physiopathology hypothesis on asthma’s protective mechanisms for death on coronavirus disease (COVID-19) infections. Atopic patients have a low production of interferons alpha (represented in red), beta (represented in orange), and gamma (represented in purple), which constitutes an inadequate antiviral defense (section 1 of the figure). In this scenario, lower interferon productions (mainly alpha) lead to a predominance of the Th2 response over the Th1 in patients with asthma (section 2 of the figure). This predominance may be protective, as it can downregulate the late phase of hyperinflammation in patients with asthma, which is a hallmark of severe respiratory viral infections (section 3 of the figure). Moreover, the inflammatory environment of the bronchoalveolar system in patients with asthma could result in a decrease in the expression of the angiotensin-converting enzyme 2 (ACE-2) (represented in blue), a known severe acute respiratory syndrome coronavirus 2 (SARS-CoV-2) binding receptor, due to interleukin-13 eosinophil recruitment (section 4 of the figure).

However, the increase in the Th2 response dominance over Th1 may be protective because it can downregulate the late phase of hyperinflammation in patients with asthma, which is a hallmark of severe respiratory viral infections (15). In patients with severe COVID-19, a high initial activation of interferons and inflammatory cytokines was described (12); the exacerbated innate immune response may be the initial trigger for the evolution of pulmonary infiltration, vascular leakage, and cytokine storm (12). Also, the high number of eosinophils in patients with asthma may thus be another protective mechanism against severe COVID-19 disease (12, 14–16). Moreover, the inflammatory environment of the bronchoalveolar system in patients with asthma could result in a decrease in the expression of the angiotensin-converting enzyme 2 (ACE-2), a known SARS-CoV-2 binding receptor, due to interleukin-13 (IL-13) eosinophil recruitment (4, 17). We presented the overview of the physiological process that induces our study hypothesis in Figure 1.

In this context, we performed an epidemiology study associating the characteristics of hospitalized patients due to severe acute respiratory syndrome (SARS) during the SARS-CoV-2 infection with the asthma diagnosis in a Brazilian cohort study.

## Materials and methods

We performed an epidemiological study using patients’ characteristics from Brazilian individuals hospitalized due to SARS during the COVID-19 pandemic with a positive real-time reverse-transcriptase polymerase chain reaction (RT-PCR) for SARS-CoV-2. We retrieved the patients’ characteristics from OpenDataSUS,^1^ and we included the following data: demographic information (e.g., sex, age, place of residence, educational level, and race), clinical signs, comorbidities, need for ICUs and mechanical ventilation support, and outcomes. The data were computed by the Brazilian Ministry of Health according to SARS surveillance data from the Information System for Epidemiological Surveillance of Influenza (SIVEP-Flu; in Portuguese, *Sistema de Informação de Vigilância Epidemiológica da Gripe*). We obtained the data between 17 February 2020 and 10 October 2021. In addition, the dataset used in the present study was published and validated in other studies (18–33).

We grouped the patients according to asthma status to compare them for all patients’ characteristics (demographic information, clinical signs, comorbidities, need for ICUs and mechanical ventilation support, and outcomes). We excluded the hospitalized patients with SARS and without confirmatory SARS-CoV-2 RT-PCR from the dataset. In addition, we removed the patients due to a lack of asthma information.

We did the statistical analysis using the Statistical Package for the Social Sciences (IBM SPSS Statistics for Macintosh, Version 27.0). We applied the Chi-square statistical test to compare the proportions of the earlier groups (patients with and without asthma) in relation to all patients’ characteristics (demographic information, clinical signs, comorbidities, need for ICUs and mechanical ventilation support, and outcomes). After that, we calculate the relative risk (RR) or odds ratio (OR) and the 95% confidence interval (95% CI) to estimate the impact that COVID-19 has on different groups, also considering all patients’ characteristics, mainly death as the outcome. We also performed one multivariate analysis using binary regression (Backward model) to identify the main predictors for death (outcome) among the patient’s characteristics, including demographic information and comorbidities. In the text of the manuscript, to summarize our findings, we decided to present our results for the inferential analysis using the OR and 95% CI for the statistical tests with a significant *P*-value. We detailed all the *P*-values for each inferential analysis in the tables and Supplementary material. We presented the data using the number of individuals (*N*) and the percentage (%). The results were summarized in tables and figures. The figures were built using the GraphPad Prism version 8.0.0 for Mac, GraphPad Software, San Diego, CA, USA.^2^ We adopted an alpha error (α) of 0.05 in all statistical analyzes.

The epidemiologic data used in this epidemiological study are public, and it does not contain personal data about the patients, which means that consent is not required.

## Results

### Epidemiologic data: Patients’ characteristics of hospitalized severe acute respiratory syndrome individuals due to severe acute respiratory syndrome coronavirus 2 infection in Brazil

**FIGURE 2:**
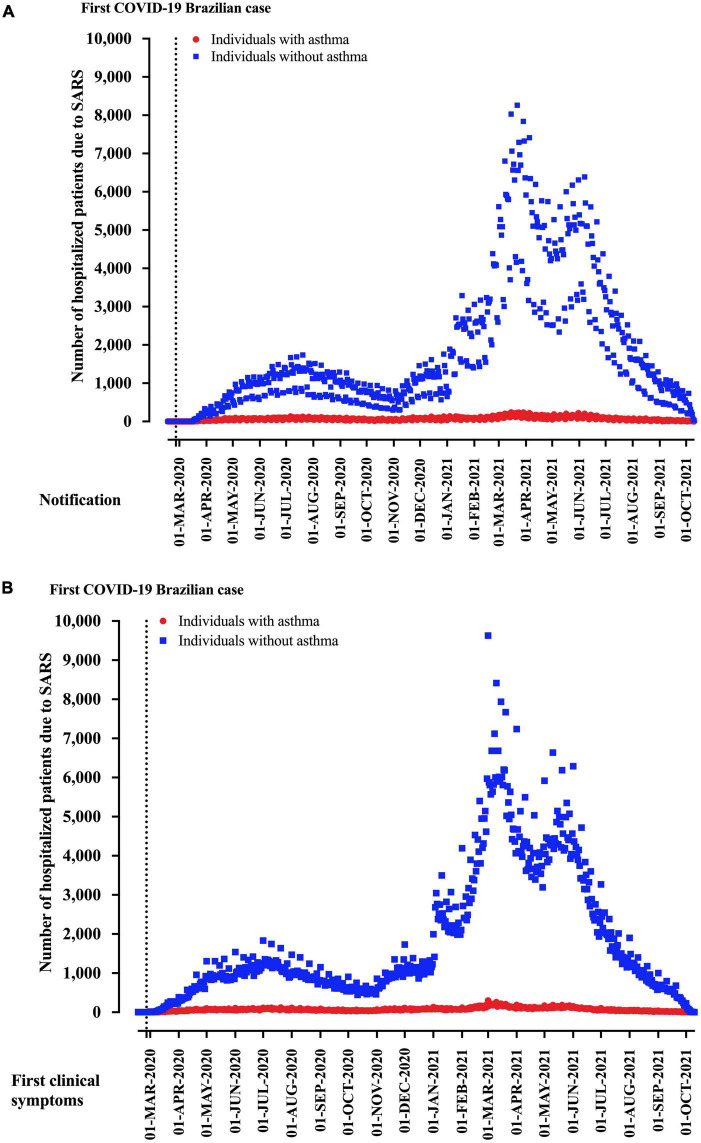
Distribution of patients hospitalized with severe acute respiratory syndrome (SARS) due to coronavirus disease (COVID-19) in Brazil according to asthma diagnosis. We presented the data distributed according to the **(A)** weeks of disease notification and **(B)** start of clinical signs.

From 2,740,272 hospitalized SARS patients, we excluded 923,174 patients due to the absence of SARS-CoV-2 infection (negative SARS-CoV-2 RT-PCR) and 687,286 due to a lack of asthma information. Finally, 1,129,838 hospitalized SARS patients due to COVID-19 and with the description for asthma status were included in the study. For the region of notification, residence, and hospitalization, São Paulo state was the most prevalent, with 29.4% of the cases, followed by the Minas Gerais state (12.3%) and Paraná state (7.4%) (Table 1). Distribution of patients hospitalized with SARS due to COVID-19 in Brazil according to asthma diagnosis is presented in Figure 2 for weeks of disease notification (Figure 2A) and weeks of start of clinical signs (Figure 2B).

**TABLE 1:** Brazil’s region of notification, residence, and hospitalization of hospitalized patients due to severe acute respiratory syndrome (SARS) by the coronavirus disease (COVID-19) and the description of the asthma phenotype.

States and federal district	Notification (%)	Residence (%)	Hospitalization (%)
Acre	1,847 (0.2)	1,868 (0.2)	1,858 (0.2)
Alagoas	12,787 (1.1)	12,852 (1.1)	12,783 (1.1)
Amazonas	21,196 (1.9)	21,770 (1.9)	21,189 (1.9)
Amapá	4,296 (0.4)	4,165 (0.4)	4,296 (0.4)
Bahia	47,754 (4.2)	47,745 (4.2)	47,584 (4.2)
Ceará	30,680 (2.7)	30,662 (2.7)	30,678 (2.7)
Federal District	29,281 (2.6)	26,853 (2.4)	29,254 (2.6)
Espírito Santo	4,745 (0.4)	4,862 (0.4)	4,747 (0.4)
Goiás	48,459 (4.3)	50,338 (4.5)	48,503 (4.3)
Maranhão	12,814 (1.1)	13,283 (1.2)	12,804 (1.1)
Minas Gerais	138,851 (12.3)	139,369 (12.3)	138,883 (12.3)
Mato Grosso do Sul	22,323 (2.0)	22,569 (2.0)	22,315 (2.0)
Mato Grosso	21,806 (1.9)	22,106 (2.0)	21,800 (1.9)
Pará	26,373 (2.3)	26,815 (2.4)	26,375 (2.3)
Paraíba	22,509 (2.0)	22,435 (2.0)	22,509 (2.0)
Pernambuco	17,220 (1.5)	17,382 (1.5)	17,393 (1.5)
Piauí	12,018 (1.1)	11,594 (1.0)	12,019 (1.1)
Paraná	83,129 (7.4)	83,149 (7.4)	83,129 (7.4)
Rio de Janeiro	74,604 (6.6)	74,806 (6.6)	74,558 (6.6)
Rio Grande do Norte	8,817 (0.8)	8,830 (0.8)	8,819 (0.8)
Rondônia	8,338 (0.7)	8,545 (0.8)	8,338 (0.7)
Roraima	2,597 (0.2)	2,633 (0.2)	2,597 (0.2)
Rio Grande do Sul	75,792 (6.7)	75,927 (6.7)	75,792 (6.7)
Santa Catarina	52,308 (4.6)	52,099 (4.6)	52,307 (4.6)
Sergipe	9,666 (0.9)	9,570 (0.8)	9,667 (0.9)
São Paulo	332,609 (29.4)	330,437 (29.3)	332,615 (29.4)
Tocantins	7,019 (0.6)	6,991 (0.6)	7,018 (0.6)

We presented the data as the number of patients (*N*) and percentage (%).

We observed a predominance of male patients (632,696; 56%) (Table 2). Considering the age, 4,939 (0.4%) patients were less than one year old (y.o.), 8,850 (0.8%) patients were between one and 12 y.o., 20,976 (1.9%) patients were between 13 and 24 y.o., 611,864 (54.2%) patients were between 25 and 60 y.o., 252,260 (22.3%) patients were between 61 and 72 y.o., 167,534 (14.8%) patients were between 73 and 85 y.o., and 63,415 (5.6%) patients were +85 y.o. (Table 2). The patients were majority Whites (504,436; 53.2%), with a multiracial background (*Pardos*) (383,566, 40.4%), and Blacks (47,864, 5.0%) (Table 2).

**TABLE 2:** Characterization of Brazilian hospitalized patients with severe acute respiratory syndrome (SARS) due to the coronavirus disease (COVID-19).

Patient’s characteristics	Group	*N* (%)
Sex	Female	496,972 (44)
	Male	632,696 (56)
Age (years old, y.o.)	0–12 y.o.	13,789 (1.2)
	13–24 y.o.	20,976 (1.9)
	25–60 y.o.	611,864 (54.2)
	61–72 y.o.	252,260 (22.3)
	73–85 y.o.	167,534 (14.8)
	+85 y.o.	63,415 (5.6)
Race	White	504,436 (53.2)
	Black	47,864 (5.0)
	Asian	10,789 (1.1)
	Multiracial background (*Pardos*)	383,466 (40.4)
	Indigenous	1,779 (0.2)
Educational level	Illiterate	27,483 (6.1)
	Middle school first cycle	119,784 (26.7)
	Middle school second cycle	84,594 (18.8)
	High school	146,574 (32.6)
	University education	65,859 (14.7)
	Does not apply	4,890 (1.1)
Place of residence	Urban	969,346 (94.5)
	Rural	52,634 (5.1)
	Peri-urban	3,286 (0.3)
Living in a flu outbreak region	Yes	79,246 (27.7)
	No	207,324 (72.3)
Nosocomial infection	Yes	18,522 (2.0)
	No	895,922 (98.0)
Clinical signs	Fever	641,034 (62.6)
	Cough	790,485 (75.6)
	Sore throat	204,933 (21.8)
	Dyspnea	824,547 (78.7)
	Respiratory discomfort	669,097 (66.4)
	SpO_2_ <95%	758,810 (74.2)
	Diarrhea	157,490 (16.8)
	Abdominal pain	67,445 (8.1)
	Fatigue	293,907 (34.1)
	Loss of smell	113,401 (13.5)
	Loss of taste	115,379 (13.8)
	Vomit	96,013 (10.4)
	Other symptoms	372,899 (41.6)
Comorbidities	Puerperal	3,057 (0.3)
	Cardiopathies	340,295 (30.5)
	Hematologic diseases	7,262 (0.7)
	Down syndrome	3,406 (0.3)
	Hepatic diseases	8,675 (0.8)
	Asthma	43,245 (3.8)
	Diabetes mellitus	241,699 (21.7)
	Neurological diseases	35,980 (3.2)
	Immunosuppressive diseases	23,784 (2.1)
	Chronic renal diseases	35,460 (3.2)
	Obesity	96,121 (8.7)
	Other comorbidities	273,913 (25.3)
Received Influenza	Yes	120,149 (22.4)
vaccine in the last campaign in Brazil	No	415,795 (77.6)
Antiviral use to treat the Flu clinical signs	Yes	68,218 (8.2)
	No	765,281 (91.8)
Thorax X-ray findings	Normal findings	18,902 (3.0)
	Infiltrate interstitial	150,000 (24.0)
	Consolidation	17,034 (2.7)
	Mixed findings	21,416 (3.4)
	Other findings	62,351 (10.0)
	Not performed	354,249 (56.8)
Thorax computerized tomography findings	COVID-19 typical findings	450,867 (65.5)
	COVID-19 undetermined	16,941 (2.5)
	COVID-19 atypical	9,020 (1.3)
	Negative for pneumonia	1,756 (0.3)
	Other findings	29,586 (4.3)
	Not performed	179,735 (26.1)
Need for intensive care unit	Yes	366,350 (36.4)
	No	640,981 (63.6)
Need for mechanical ventilatory support	Invasive	207,086 (20.8)
	Non-invasive	604,467 (60.7)
	Non-required*	184,940 (18.6)
Closure criteria	Laboratory criterion	1,000,501 (91.0)
	Clinical epidemiological findings	11,080 (1.0)
	Clinical findings	24,669 (2.2)
	Clinical and images findings	63,058 (5.7)
Patient’s evolution (outcome)	Cure	679,172 (65.8)
	Death	350,496 (34.0)
	Non-related death to SARS	2,240 (0.2)
Received the	Yes	124,087 (28.1)
SARS-CoV-2 vaccine in Brazil	No	317,535 (71.9)

We presented the data as the number of patients (*N*) and percentage (%); SpO_2_, arterial oxygen saturation. *In some cases, mechanical ventilation was not performed due to a lack of equipment.

For educational level, the patients had mainly high school level (146,574; 32.6%), followed by the first (119,784; 26.7%) and second (84,594; 18.8%) cycles of elementary school. Only 65,859 (14.7%) patients had a superior level of education, and 27,483 (6.1%) were illiterates (Table 2). The patients lived majority in urban areas (969,346; 94.5%), and the frequency of patients living in Flu outbreak regions was low (79,246; 27.7%) (Table 2).

The main clinical signs among the SARS patients due to SARS-CoV-2 infection were dyspnea (824,547; 78.7%), cough (790,485; 75.6%), arterial oxygen saturation (SpO_2_) <95% (758,810; 74.2%), and respiratory discomfort (669,097; 66.4%) (Table 2). In addition, the main comorbidities in this group of patients were the presence of cardiopathies (340,295; 30.5%), diabetes mellitus (241,699; 21.7%), obesity (96,121; 8.7%), kidney diseases (35,460; 3.2%), neurologic diseases (35,980; 3.2%), and immunosuppressive diseases (23,784; 2.1%) (Table 2). Curiously, only 43,245 (3.8%) patients had asthma.

Most patients submitted to thorax X-rays in imaging data analysis had infiltrate interstitial (150,000; 24%). In contrast, most patients submitted to high-resolution computed tomography of the chest showed typical COVID-19 images (450,867; 65.5%) (Table 2). Upon ventilatory support requirements, most patients were under non-invasive mechanical ventilation (604,467; 60.7%), followed by those requiring invasive mechanical ventilation (207,086; 20.8%). Finally, only 184,940 (18.6%) patients did not require mechanical ventilation support, or mechanical ventilation was not performed due to a lack of equipment (Table 2). For discharge criteria, 1,000,501 (91.0%) patients were discharged upon laboratory findings, 11,080 (1.0%) based on clinical epidemiological findings, 24,669 (2.2%) based on clinical findings, and 63,058 (5.7%) based on clinical and images findings (Table 2). The main outcome in our study population was clinical recovery (679,172; 65.8%), followed by death due to COVID-19 (350,496; 34.0%) and due to non-COVID-19 related causes (2,240; 0.2%). Finally, among the patients included after the vaccination onset, only 124,087 (28.1%) were vaccinated for COVID-19 (against SARS-CoV-2 infection) (Table 2).

### Association between the patient’s characteristics and the asthma phenotype

**FIGURE 3:**
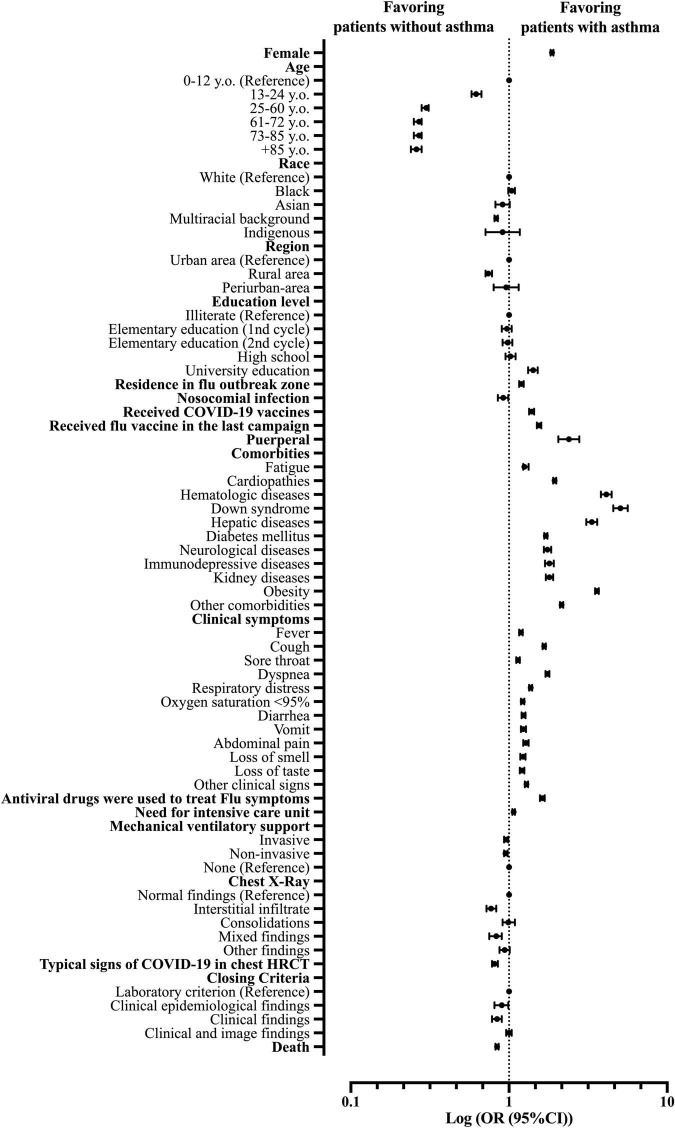
Patients’ characteristics associated with the asthma phenotype in patients hospitalized with severe acute respiratory syndrome (SARS) due to coronavirus disease (COVID-19) in Brazil. OR, odds ratio; 95% CI, 95% confidence interval; HRCT, high-resolution computed tomography of the chest.

The female sex was more frequent in patients with asthma compared to non-asthmatic patients (OR = 1.87; 95% CI = 1.83–1.91). There were no significant differences between age groups for patients with asthma and non-asthmatic patients, and the patients aged between 25 and 60 y.o. were the most prevalent for both groups (54.2%). As for race comparison, the white race was more prevalent in patients with asthma than in non-asthmatic; the same was for the Black race (OR = 1.04; 95% CI = 0.99–1.09), while multiracial background patients (*Pardos*) were more frequent in the non-asthmatic group (OR = 0.83; 95% CI = 0.81–0.85). Indigenous and Asian patients had the same frequency in both groups (0.2 and 1.1%, respectively). For patients’ education levels, our data demonstrated that patients with asthma were more prone to have higher education levels, mainly at the college level (OR = 1.42; 95% CI = 1.32–1.52). Nonetheless, patients with asthma were more likely to live in a Flu outbreak region (OR = 1.20; 95% CI = 1.16–1.24) (Supplementary Table 1 and Figure 3).

The clinical signs were more frequent in patients with asthma compared to non-asthmatic patients for fever [66.4 vs. 62.5% (OR = 1.1; 95% CI = 1.16–1.22)], cough [81.2 vs. 75.4% (OR = 1.67; 95% CI = 1.63–1.71)], sore throat [24.0 vs. 21.7% (OR = 1.14; 95% CI = 1.11–1.17)], dyspnea [86.4 vs. 78.4% (OR = 1.75; 95% CI = 1.70–1.80)], respiratory discomfort [72.9 vs. 66.2% (OR = 1.37; 95% CI = 1.34–1.40)], SpO_2_ <95% [77.7 vs. 74.1% (OR = 1.22; 95% CI = 1.19–1.25)], diarrhea [19.9 vs. 16.7% (OR = 1.24; 95% CI = 1.20–1.27)], abdominal pain [10.0 vs. 8.0% (OR = 1.28; 95% CI = 1.23–1.33)], fatigue [39.2 vs. 34.0% (OR = 1.25; 95% CI = 1.23–1.33)], loss of smell [16.0 vs. 13.4% (OR = 1.23; 95% CI = 1.18–1.27)]; loss of taste [16.0 vs. 13.4% (OR = 1.21; 95% CI = 1.17–1.25)], vomit [12.4 vs. 10.3% (OR = 1.23, 95% CI = 1.19–1.28)], and other clinical signs [47.6 vs. 41.6% (OR = 1.29; 95% CI = 1.26–1.32)] (Supplementary Table 2 and Figure 3).

We observed that patients with asthma were more likely to present other comorbidities alongside asthma than non-asthmatic patients upon COVID-19 diagnosis like cardiopathies [45.4 vs. 30.0% (OR = 1.94; 95% CI = 1.90–1.99)], hematologic diseases [2.5 vs. 0.6% (OR = 4.12; 95% CI = 3.81–4.46)], Down syndrome [1.4 vs. 0.3% (OR = 5.07; 95% CI = 4.56–5.63)], hepatic diseases [2.4 vs. 0.7%, (OR = 3.34; 95% CI = 3.08–3.61)], diabetes mellitus [31.7 vs. 21.4% (OR = 1.71; 95% CI = 1.67–1.75)], neurological diseases [5.4 vs. 3.2% (OR = 1.75; 95% CI = 1.66–1.85)], immunosuppressive diseases [3.7 vs. 2.1% (OR = 1.80; 95% CI = 1.69–1.92)], kidney diseases [5.5 vs. 3.1% (OR = 1.80; 95% CI = 1.71–1.90)], obesity [24.6 vs. 8.3% (OR = 3.60; 95% CI = 3.50–3.70)], and other comorbidities [41.6 vs. 24.9% (OR = 2.15; 95% CI = 2.10–2.20)] (Supplementary Table 3 and Figure 3).

Nonetheless, patients with asthma were more likely to receive the Influenza virus vaccine in the last campaign than non-asthmatic patients [30.5 vs. 22.1% (OR = 1.55; 95% CI = 1.50–1.60)]. These patients were also more likely to use antivirals drugs to treat clinical signs [12.4 vs. 8.0% (OR = 1.63; 95% CI = 1.57–1.68)], were more prompted to require intensive care support [37.8 vs. 36.3% (OR = 1.07; 95% CI = 1.05–1.09)] and were more likely to have had COVID-19 vaccine against SARS-CoV-2 infection [34.9 vs. 27.9% (OR = 1.39; 95% CI = 1.34–1.44)]. The results were similar regarding mechanical ventilation requirements between patients with asthma and non-asthmatic patients for invasive, non-invasive, and not required mechanical ventilatory support (or mechanical ventilation was not performed due to a lack of equipment) (Supplementary Table 4 and Figure 3). In contrast, non-asthmatic patients were more likely to evolve to death compared to patients with asthma [30.3% of the patients with asthma evolved to death while 34.2% of non-asthmatic patients had the same outcome (OR = 0.84; 95% CI = 0.82–0.86)] (Supplementary Table 4 and Figure 3).

### Association between outcome and the asthma diagnosis in hospitalized patients with severe acute respiratory syndrome due to coronavirus disease in Brazil, according to the mechanical ventilation support

Among the patients with asthma, 74.7% who required invasive ventilatory support (or mechanical ventilation was not performed due to a lack of equipment) evolved to death. In contrast, 78.0% of non-asthmatic patients evolved to death (OR = 0.83; 95% CI = 0.79–0.88). Also, 20.0% of the patients with asthma that required non-invasive ventilatory support evolved to death, while 23.5% of non-asthmatic patients evolved to death (OR = 0.81; 95% CI = 0.79–0.84). Finally, only 11.2% of the patients with asthma who did not require any ventilatory support evolved to death, while 15.8% of non-asthmatic patients evolved to death (OR = 0.67; 95% CI = 0.62–0.72) (Table 3).

**TABLE 3:** Association between outcome and the asthma diagnosis in hospitalized patients with severe acute respiratory syndrome (SARS) due to coronavirus disease (COVID-19) in Brazil, according to the mechanical ventilation support.

Mechanical ventilation support	Group*	Asthma		Total (%)	*P*-value	OR	95% CI
						
		Yes (%)	No (%)				
All patients	Cure	27,719 (69.7)	651,453 (65.8)	679,172 (66.0)	<0.001	1	Reference
	Death	12,053 (30.3)	338,443 (34.2)	350,496 (34.0)	–	0.84	0.82–0.86
Invasive	Cure	1,962 (25.3)	41,886 (22.0)	43,848 (22.2)	<0.001	1	Reference
	Death	5,798 (74.7)	148,267 (78.0)	154,065 (77.8)	–	0.83	0.79–0.88
Non-invasive	Cure	17,131 (80.0)	404,197 (76.5)	421,328 (76.6)	<0.001	1	Reference
	Death	4,289 (20.0)	124,421 (23.5)	128,710 (23.4)	–	0.81	0.79–0.84
Non-required**	Cure	6,056 (88.8)	134,184 (84.2)	140,240 (84.3)	<0.001	1	Reference
	Death	765 (11.2)	25,269 (15.8)	26,034 (15.7)	–	0.67	0.62–0.72

We presented the data as the number of patients (*N*) and percentage (%). We did the statistical analysis using the Chi-square test and used an alpha error of 0.05. OR, odds ratio; 95% CI, 95% confidence interval. *Patients with deaths unrelated to SARS were excluded from this statistical analysis. **In some cases, mechanical ventilation was not performed due to a lack of equipment.

### Multivariate analysis to identify the predictors for death in hospitalized patients with severe acute respiratory syndrome due to coronavirus disease in Brazil

**FIGURE 4:**
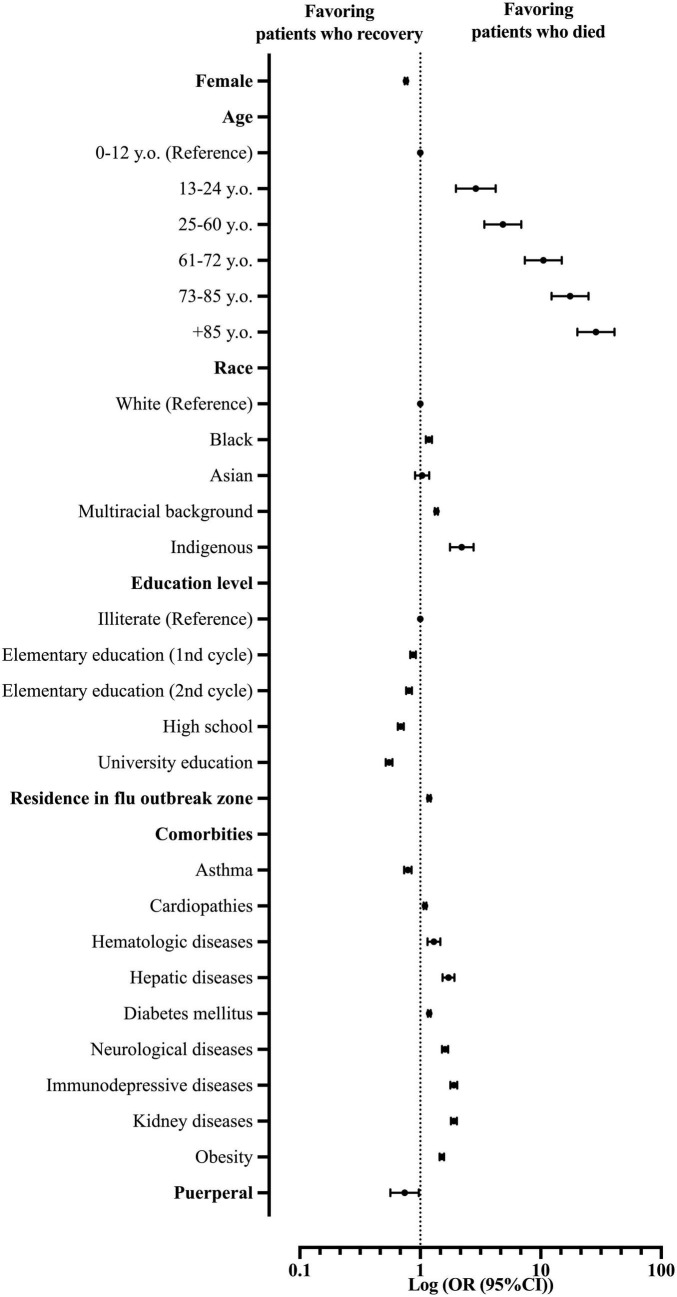
Multivariate analysis demonstrates the main predictors for death in patients hospitalized with severe acute respiratory syndrome (SARS) due to coronavirus disease (COVID-19) in Brazil. OR, odds ratio; 95% CI, 95% confidence interval.

Our multivariate model using the Logistic Regression (Backward model) was able to predict the chance of death among hospitalized patients with SARS due to COVID-19 in Brazil (*R*^2^ of Cox and Snell = 0.129; *R*^2^ of Nagelkerke = 0.176; *P*-value < 0.001). Patient’s characteristics included in step one: sex, age, race, educational level, comorbidities, place of residence, and living in a Flu outbreak region. In the analyses, we did not consider Down syndrome and the place of residence as predictors (Supplementary Table 5 and Figure 4).

From the patients’ characteristics, the female patients were less prone to die from COVID-19 compared to the male patients (OR = 0.76; 95% CI = 0.74–0.78). In this scenario, older age also plays a risk of death: 13–24 y.o. (OR = 2.89; 95% CI = 1.97–4.23); 25–60 y.o. (OR = 4.84; 95% CI = 3.40–6.89); 61–72 y.o. (OR = 10.51; 95% CI = 7.39–14.95); 73–85 y.o. (OR = 17.51; 95% CI = 12.31–24.91); and +85 y.o. (OR = 28.70; 95% CI = 20.14–40.92). Race also plays a role in the risk of death in the SARS-CoV-2 infection. Indigenous patients were more prone to die (OR = 2.21; 95% CI = 1.77–2.76) compared to White patients, followed by patients with a multiracial background (*Pardos*) (OR = 1.36; 95% CI = 1.32–1.40) and Blacks (OR = 1.18; 95% CI = 1.11–1.25). Interestingly, the higher the patient’s education level, the less prone they would die from the infection. Compared to illiterate patients, patients with college educations were less prone to die (OR = 0.55; 95% CI = 0.52–0.59), followed by patients with high school education (OR = 0.69; 95% CI = 0.65–0.73), second cycle middle school education (OR = 0.81; 95% CI = 0.76–0.85), and first cycle middle school (OR = 0.87; 95% CI = 0.83–0.92). Nonetheless, living in a Flu outbreak region presents a higher risk for death due to SARS-CoV-2 infection (COVID-19) (OR = 1.18; 95% CI = 1.15–1.22) in our study population (Supplementary Table 5 and Figure 4).

As for patients’ comorbidities, the majority of conditions presented themselves as risk factors for death due to SARS-CoV-2 infections, such as immunosuppressive diseases (OR = 1.90; 95% CI = 1.78–2.03), kidney diseases (OR = 1.90; 95% CI = 1.79–2.01), hepatic diseases (OR = 1.72; 95% CI = 1.53–1.92), neurological diseases (OR = 1.61; 95% CI = 1.52–1.70), obesity (OR = 1.51; 95% CI = 1.44–1.57), hematologic diseases (OR = 1.30; 95% CI = 1.15–1.47), diabetes mellitus (OR = 1.19; 95% CI = 1.16–1.22), and cardiopathies (OR = 1.09; 95% CI = 1.06–1.12). Curiously, one comorbidity and one patient’s characteristic stood out as protective factors against death in SARS-CoV-2 infection (COVID-19). Patients with asthma were less prone to die compared to other patients (OR = 0.79; 95% CI = 0.73–0.85), just like puerperal patients compared to other patients (OR = 0.74; 95% CI = 0.56–0.97) (Supplementary Table 5 and Figure 4).

## Discussion

Considering the results that we obtained, the ones that most stood out to us were associated with asthma disease. Our results indicated that patients with asthma developed more symptoms but died less than patients without the disease. In the multivariate analysis, asthma returned as a protective factor against death due to COVID-19. Other relevant results discussed include the COVID-19 disease incidence in Brazil by sex, age, race, and education, and puerperium as a protective factor against death.

According to our data, COVID-19 was more prevalent in males, Whites, 25–60 y.o. populations who had completed high school. In a study made in Wuhan, China, males were more affected by the disease, although the most prevalent age group was between 50 and 59 y.o. (34). In the multivariate analysis, the male sex was a risk factor for the mortality of people infected by COVID-19 and age, with older ages being related to a worse prognosis, in agreement with other studies found in the literature, including a meta-analysis of 212 studies and 281,461 individuals (35). Indigenous peoples were more prone to die from COVID-19 than other races, followed by multiracial background patients (*Pardos*) and the Black race, and then by White patients (19, 33, 36, 37). Interestingly, our data demonstrated a relationship between education level and mortality for COVID-19 infections, in which the higher the patients’ education level, the lower the mortality rates. These epidemiological differences can be justified by the socioeconomic differences that come with higher education related to better healthcare access, self-care, and the economic possibility of social isolation (38).

Disparities in the prevalence and clinical evolution of male and female patients can be observed in several bacterial, viral, fungal, and even parasitic infectious diseases at all ages. Other coronavirus outbreaks, such as the SARS Coronavirus 1 (SARS-CoV-1) and the Middle East Respiratory Syndrome (MERS) outbreaks, showed similar results as the present pandemic (39, 40). Innate and adaptive endocrinological and immunological differences appear to be the main related mechanisms (32, 39). Among the adaptive responses, female patients have a more significant amount of cluster of differentiation 4 (CD4)+ T cells, a cellular activity of cluster of differentiation 8 (CD8)+ T cells, and even greater antibody production than male patients (32, 39, 41). Female patients also produce more type 1 interferon, a potent antiviral cytokine. The estradiol hormone also generates advantages against infections, increasing the production of antibodies, T-lymphocytes, monocytes, neutrophils, and macrophages (39). Men also have more comorbidities associated with them and can present higher concentrations of ACE2 receptors in the lungs than women (40, 42). Furthermore, differences in the cytokine profile can be of importance, and men have higher concentrations of chemokines and cytokines such as interleukin (IL)-8, IL-18, and Chemokine (C-C motif) ligand 5 (CCL5), which correlated with higher non-classical monocytes and a worse disease progression (41). It is important to note that simple protective habits, such as washing your hands after going to the bathroom or less exposure, are more common in females, and another factor in explaining or at least contributing to the protection that women have against infectious diseases, but that fact did not justify the mortality faced whenever compared to males and females already infected by the virus (43). Despite their lower mortality (32), female patients have a more significant correlation with the development of post-COVID syndrome (43).

As several studies and ours demonstrate, age can be considered the leading independent risk factor for mortality in COVID-19 (31, 34, 44–46). The main reasons for this include immunosenescence and the ability to contribute to the cytokine storm characteristic of COVID-19 (44). Aging is characterized by a chronic pro-inflammatory state with activation and persistence of innate autoimmune activation that can increase tissue damage caused by infections in the elderly (47). This pro-inflammatory state could increase inflammatory responses leading to the cytokine storm for which the disease is known, also influencing the expression of ACE2 receptors, facilitating the entry of the virus into the patient’s system (44, 47). In addition, the reserve of the lungs and other organs is reduced, leading to the fragility of new infections (47).

Socioeconomic factors such as race, education level, and income are associated with a greater or lesser chance of mortality from COVID-19. In an epidemiological study carried out in the United States of America, Black and American Indian people were more likely to contract and die from the COVID-19 disease due to several factors, such as access to medical care, income, housing conditions with more people per household, and type of professional activity employed with a need for greater interpersonal contact (48, 49). We can use the same logic for the Black, Indigenous, and mixed-race (*Pardos*, patients with multiracial background) Brazilian population (19, 36, 37).

### Asthma and coronavirus disease

#### Initial considerations

The prevalence of asthma in the Brazilian population is around 10% (4). However, in our data, we found out that only 3.8% of the population infected with SARS-CoV-2 had asthma as a comorbidity. Also, we were able to verify that some associated comorbidities were more frequent in patients with asthma than in non-asthmatic patients. Among these comorbidities, in our data, immunosuppressive diseases, kidney diseases, hepatic diseases, neurological diseases, obesity, hematologic diseases, diabetes mellitus, and cardiopathies were associated with a higher risk of death. These data lead to the possibility of comorbidities being associated with the asthma phenotype for the patient to develop these conditions, which are corroborated by several studies that entangle such comorbidities with the asthma phenotype, resulting in difficulty in controlling the asthma phenotype, higher exacerbations, and more hospitalizations (50–52). Nonetheless, a meta-analysis of eleven studies with 117,548 patients with asthma compared with 443,948 non-asthma controls showed an intimate association between asthma and higher comorbid conditions, such as obesity, hypertension, diabetes mellitus, cardiopathies, and cerebrovascular disorders (53). The study also concluded that respiratory comorbidities are five times more prevalent in asthma than in non-asthma patients and evaluated that management of comorbidities in asthma control strategies can improve disease outcomes (53).

#### Patients with asthma developed more clinical signs than patients without the disease

Patients with asthma developed a greater number of clinical signs compared to non-asthmatic patients. Asthma causes respiratory symptoms such as dyspnea, coughing, wheezing, and chest discomfort. The intensity and frequency of symptoms can vary from individual to individual or even in the same patient when considering different year periods (54, 55). In addition, those symptoms mentioned can be attributed to cases of COVID-19 in different incidences and accompanied by other symptoms not characteristic of asthma. It is possible that the appearance of these symptoms in common more frequently is due to the stress that a viral infection can cause to a patient, generating an overlap of the two pathophysiological processes. In addition, it is essential to remember that most patients with asthma who presented symptoms had other associated comorbidities, according to the data collected.

Due to the pandemic being still very recent, most of the more elaborate articles are still being developed, and therefore there’s a shortage of data. Still, according to the U.S. Centers for Disease Control and Prevention (CDC), individuals with asthma are also at higher risk for hospitalization and other severe outcomes from COVID-19 (56). In this manner, a study was conducted on 436 patients with COVID-19 admitted to the University of Colorado Hospital and described a non-significant association between asthma diagnosis and greater intubation odds among patients with COVID-19, even after adjusting for body mass index and age, which are well-known risk factors for severity, and were significantly associated with intubation in their model (57). Nonetheless, a meta-analysis study with data from 57 articles published on electronic databases, including preprint repositories and the World Health Organization (WHO) COVID-19 database until 26 May 2020, showed the prevalence of asthma among those infected with COVID-19 of 7.46% (2). Moreover, a pooled analysis showed a 14% risk ratio reduction in acquiring COVID-19 and a 13% reduction in hospitalization with COVID-19 for people with asthma compared with those without (2). There was no significant difference in the combined risk of requiring admission to the ICU or receiving mechanical ventilation for people with asthma and the risk of death from COVID-19. Sunjaya et al. (2) concluded that people with asthma have a lower risk than those without asthma of acquiring COVID-19 and have similar clinical outcomes (2).

Another meta-analysis study conducted with 131 studies (410,382 patients) found no significant difference in asthma prevalence between hospitalized and non-hospitalized, severe and non-severe, ICU and non–ICU, intubated/mechanically ventilated and non-intubated/mechanically ventilated patients with COVID-19 (58). Furthermore, the study stated that asthma is not associated with higher intubation or mechanical ventilation risk. In contrast, the study concluded that patients with asthma have a lower risk of death than patients without asthma (58), corroborating our data.

#### Asthma patients died less from coronavirus disease

Patients with asthma died less from COVID-19 than patients with no comorbidities, standing as a protective factor against the disease caused by the new Coronavirus. This result, although suspected by our group, may initially be seen as a contradiction in the literature since viruses are etiological agents widely known for their ability to trigger attacks in patients with asthma, both children and adults, the main etiological agents being Respiratory Syncytial Virus and Rhinovirus (50–61). The immune response in viral infections is characterized by the initial activation of innate immunity with the production of IFN-α, IFN-β, and IFN-γ, which are responsible for containing the viral spread (14–16). Next, cells and dendritic cells are responsible for the increased production of peripheral IFN-α (14–16). Interestingly, atopic patients have low interferon production, constituting a deficient antiviral defense. Interferons act as negative regulators of the Th2 response. There is a link between IgE and IFN-α, as IgE downregulates TLR-9, reducing interferon production by dendritic cells (14–16). In this context, the antiviral response would be impaired in atopic patients (14–16).

In the Coronavirus-family Infections, cases with a worse prognosis are associated with high initial activation of interferons and inflammatory cytokines (12, 14–16). Exacerbated innate immune response may trigger evolution with pulmonary infiltration, vascular leakage, and cytokine storm (12, 14–16). Considering that atopic patients have a lower Th2-related response, this could be one of the reasons why patients with asthma have a better prognosis for COVID-19 than healthy patients (12, 14–16). In addition, patients with COVID-19 typically have reduced circulating eosinophils (12, 14–16). It is also speculated that the high number of eosinophils in patients with asthma may thus be another protective mechanism (12, 14–16).

Nonetheless, another possible mechanism involved in the protection of patients with asthma is the inflammatory micro- and macro-environment created by the pathological process that would reduce the expression of ACE-2 receptors that would suffer downregulation due to the expression of IL-13 secreted by local eosinophils, cells related to the pathophysiological process of asthma as already exposed and with the recovery of patients who were contaminated by SARS-CoV-2 (4, 17).

Patients with asthma appeared to perform greater health care than non-asthmatic patients. In our results, patients with asthma were more likely to vaccinate themselves against the Influenza virus and the new Coronavirus. This information should be considered since more attentive clinical care, and a higher vaccination rate than the general population may have overestimated the protective factor of asthma.

A study published in June 2020 described the Mount Sinai Health System (MSHS) COVID-19 registry results to determine the prevalence of asthma and the association between a history of asthma and mortality (62). The study concluded there was no statistically significant association between asthma status and mortality among patients with COVID-19 (62). Moreover, multivariable logistic regression analysis adjusted for age, sex, race, and COVID-19 status showed that asthma was not associated with a higher risk of mortality in the entire sample or among patients who tested positive for COVID-19 with a history of asthma (62).

In another study conducted at Stanford Health Care (SHC), asthma was not an independent risk factor for hospitalization. Among SARS-CoV-2-positive patients with asthma, allergic asthma lowered the risk of hospitalization and had a protective effect compared with non-allergic asthma, showing similar results as our data (63). Curiously, in the inpatient sub-cohort followed longitudinally, patients with asthma and non-asthmatics had similar time to resolution of COVID-19 symptoms, notably lower respiratory symptoms (63) when our data showed that although patients with asthma died less from COVID-19 infections, they developed more clinical symptoms than non-asthmatic patients.

A meta-analysis from June 2021 included 11 studies with 6,046 patients (64), and the authors did not demonstrate an association with poor composite outcomes. Furthermore, subgroup analysis showed that asthma was not associated with severe COVID-19, mortality, or poor outcomes (64).

#### Th1 and Th2-related response in asthma: Role in the coronavirus disease

Th1 and Th2 cells have been the topic of the most intense study regarding asthma’s mechanisms, with the Th1 elaborating IFN-γ, interleukin (IL)-2, and lymphotoxin, and the Th2 elaborating interleukins (IL)-4, IL-5, IL-9, IL-10, and IL-13 (65, 66). Both Th1 and Th2 cells are formed from a common naive precursor T cell and differentiate into polarized populations based on signals from the local microenvironment (65, 67). In the presence of CD8α+ dendritic cells and/or interleukin (IL)-12, IL-18, or IFN-γ, they differentiate into Th1 cells. In the presence of CD8α–dendritic cells and/or IL-4 (which can come from IgE-activated mast cells or dendritic cells), Th2 cells are formed (65, 67).

Interestingly, Th1/Th2 counter-regulation has also been described, with each cell population inhibiting and/or regulating the development and/or phenotype induced by the other (65, 68, 69). The production of IL-4 and IL-10 by Th2 cells blocks the production of cytokines by Th1 and natural killers (NK) cells. Th1 cells, by secreting IFN-γ, inhibit the proliferation and differentiation of basophils, mastocytes, and eosinophils, whose activities are controlled by the Th2 synthesis of interleukins (IL)-3, IL-4, IL-5, and IL-10 (65, 68, 69). There is considerable evidence of the role of Th2 cells in the pathogenesis of asthma, but very little is known about the mechanisms determining the aberrant expansion of Th2 cells within asthmatic airways (65, 67, 68). The number of cells expressing Th2 cytokine mRNA is increased during allergic inflammation. In some cases, CD4+ T cells from atopic patients display an aberrant *in vitro* production of IL-4 and IL-5, even in response to antigens that usually elicit Th1 responses (65, 68, 69). Treating animals with the Th1 cytokine IFN-γ decreases eosinophil recruitment during allergic inflammation. Thus, molecules capable of decreasing IgE levels and Th2 cytokine production and increasing Th1 cytokine production can inhibit allergic reactions (65, 67–69).

Several studies have shown that Th1 cytokine profile responses are more prominent during severe acute cases of COVID-19 (70, 71). Pavel et al. (70) suggest that the imbalance between Th2/Th1 cytokine profiles in the airways is associated with risk factors for developing the severe form of the disease, such as age, sex, higher amount of ACE2 receptors, and smoking (70). What seems to happen; however, is that Th1 concentrations appear to be unchanged during COVID-19 disease; however, Th2 concentrations would be high, generating the imbalance (70). In asthma, the Th2/Th1 imbalance observed in those who died from COVID-19 suggests that the stability of the Th2 pathway may be masking Th1 immunity, which is typically the main pathway in patients with asthma. This approach suggests the importance of Th1 activity in combating COVID-19 infection (70). In brief, the role of Th1/Th2 response can be a target to determine the outcomes among those with asthma and COVID-19, mainly severe ones. However, the interaction of the immunological pathways in patients with COVID-19 and asthma can be conflicting. The role of the Th2 response and its relationship with the Th1 response still remains unclear regarding the prognosis of COVID-19 (70, 71).

### Puerperium women die less from coronavirus disease

The puerperium in our analyses returned as a protective factor for the disease’s mortality caused by the new Coronavirus. Despite this, the literature seems to disagree on whether these periods would bring protection or a high risk of death from the new Coronavirus (72, 73). This period is characterized by the gradual return of the woman’s body functions before pregnancy. It is accompanied by hormonal and immunological changes that decrease the concentration of defense cells and inflammatory cytokines (72, 73). Since the pathophysiological process of COVID-19 is related to an inflammatory storm, the changes could be beneficial in reducing this stimulus, which would provide a protective factor against death during the disease.

A study performed in Mexico in the first year of the pandemic observed that the Maternal Mortality Ratio in Mexico increased by over 60% in one year during the pandemic and that COVID-19 was linked to 25.4% of maternal deaths in the studied period (74). Lethality among pregnant women diagnosed with COVID-19 was 2.8%. At the same time, asthma and immune impairment increase the propensity for developing pneumonia, obesity, and diabetes, increasing the odds of in-hospital death (74).

A Brazilian study conducted during the first few months of the pandemic, from 26 February 2020, until 7 May 2020, identified 20 COVID-19-related maternal deaths aged from 20 to 43 y.o. (75). Symptoms onset was reported during pregnancy for 12 cases, postpartum for three cases, and during the cesarean section for one case (75). In 16 cases, death occurred in the postpartum period (75). At least one comorbidity or risk factor was present in 11 cases, with asthma being the most common (75). Statistical disparities could explain the epidemiologic difference between this study and ours regarding the period and data analyzed since our study comprehend a larger period and a large spectrum of patients. In this sense, a deeper investigation with a more extensive study is indicated to elucidate this further.

### Limitations

Unfortunately, we did not have access to asthma phenotyping and asthma control assessment, and which patients were using inhaled corticosteroids once maintenance therapy can provide some degree of protection against SARS-CoV-2 infection. In addition, the dataset was not specifically built to analyze patients with asthma; in this context, some information important to understanding the asthma phenotype is not available to be evaluated, such as the smoking history. In addition, more studies on this topic should be performed to test our findings because we notably found better survival in patients with asthma than non-asthmatic patients with COVID-19 despite many comorbidities. Still, the same study population had a higher social level, better use of therapies (antiviral) vaccines, and probably was richer than the non-asthmatic people. All features also can increase survival. In brief, the response of patients with asthma against COVID-19 should be multifactorial, and we must understand the components of this clinical response one step at a time.

## Conclusion

Asthma stands as a protective factor against death in hospitalized patients with COVID-19 in Brazil. We formulated those atopic patients have a low production of interferons, which constitutes an inadequate antiviral defense. In this scenario, lower interferon productions predominate Th2 response over Th1 in patients with asthma. This predominance may be protective, as it can downregulate the late phase of hyperinflammation in patients with asthma, which is a hallmark of severe respiratory viral infections. Moreover, the inflammatory environment of the bronchoalveolar system in patients with asthma could result in a decrease in the expression of the ACE-2, a known SARS-CoV-2 binding receptor, due to IL-13 eosinophil recruitment. Despite the study’s limitations on patients’ asthma phenotype information and corticosteroid usage, this study brings to light information regarding a prevalent condition that was considered a risk factor for death in COVID-19, being ultimately protective.

## Data availability statement

The original contributions presented in this study are included in the article/Supplementary material, further inquiries can be directed to the corresponding author. The complete data can be achieved at https://opendatasus.saude.gov.br/.

## Ethics statement

Ethical review and approval was not required for the study on human participants in accordance with the local legislation and institutional requirements. Written informed consent from the patients/participants OR patients/participants legal guardian/next of kin was not required to participate in this study in accordance with the national legislation and the institutional requirements.

## Author contributions

FV, RB, and FM: data collection. NS, AP, and FM: data validation and statistical analysis. All authors wrote the manuscript, approved it, and agreed with its submission to the journal.

## Footnotes

1
https://opendatasus.saude.gov.br/

2
http://www.graphpad.com
