# Supplementary material for: Profile of coronavirus disease enlightened asthma as a protective factor against death: An epidemiology study from Brazil during the pandemic
Source: Front Med (Lausanne). 2022 Nov 29;9:953084. doi: 10.3389/fmed.2022.953084 (PMC9745079; doi:10.3389/fmed.2022.953084)
Supplement: Supplementary file 1 [file Data_Sheet_1.docx]

**Title:** Profile of coronavirus disease enlightened asthma as a protective factor against death: An epidemiology study from Brazil during the pandemic

**Running title:** Asthma and COVID-19

**Authors:** Nathalia Mariana Santos Sansone; Felipe Eduardo Valencise; Rafael Fumachi Bredariol; Andressa Oliveira Peixoto; Fernando Augusto Lima Marson

**Institution:** São Francisco University; Postgraduate Program in Health Science; Laboratory of Cell and Molecular Tumor Biology and Bioactive Compounds and Laboratory of Human and Medical Genetics.

| **Supplementary Table 1.** Association between demographic data and the asthma diagnosis in hospitalized patients with severe acute respiratory syndrome (SARS) due to coronavirus disease (COVID-19) in Brazil. | | | | | | | |
| --- | --- | --- | --- | --- | --- | --- | --- |
| **Patient’s characteristics** | **Group** | **Asthma** | | **Total** | **P-value** | **OR** | **95% CI** |
|  |  | **Yes** | **No** |  |  |  |  |
| Sex | Female | 25,472 (58.9%) | 471,500 (43.4%) | 496,972 (44.0%) | <0.001 | 1.87 | 1.83-1.91 |
|  | Male | 17,767 (41.1%) | 614,929 (56.6%) | 632,696 (56.0%) | - | 1 | Reference |
| Age (years old, y.o.) | 0-12 y.o. | 1,626 (3.8%) | 12,163 (1.1%) | 13,789 (1.2%) | - | 1 | Reference |
|  | 13-24 y.o. | 1,605 (3.7%) | 19,371 (1.8%) | 20,976 (1.9%) | <0.001 | 0.62 | 0.58-0.67 |
|  | 25-60 y.o. | 23,454 (54.2%) | 588,410 (54.2%) | 611,864 (54.2%) | <0.001 | 0.30 | 0.28-0.31 |
|  | 61-72 y.o. | 8,683 (20.1%) | 243,577 (22.4%) | 252,260 (22.3%) | <0.001 | 0.27 | 0.25-0.28 |
|  | 73-85 y.o. | 5,764 (13.3%) | 161,770 (14.9%) | 167,534 (14.8%) | <0.001 | 0.27 | 0.25-028 |
|  | +85 y.o. | 2,113 (4.9%) | 61,302 (5.6%) | 63,415 (5/6%) | <0.001 | 0.26 | 0.24-0.28 |
| Race | White | 20,091 (56.9%) | 484,345 (53.0%) | 504,436 (53.2%) | - | 1 | Reference |
|  | Black | 1,985 (5.6%) | 45,879 (5.0%) | 47,864 (5.0%) | 0.081 | 1.04 | 0.99-1.09 |
|  | Asian | 392 (1.1%) | 10,397 (1.1%) | 10,789 (1.1%) | 0.070 | 0.91 | 0.82-1.01 |
|  | Multiracial background (*Pardos*) | 12,774 (36.2%) | 370,692 (40.6%) | 383,466 (40.4%) | <0.001 | 0.83 | 0.81-0.85 |
|  | Indigenous | 65 (0.2%) | 1,714 (0.2%) | 1,779 (0.2%) | 0.517 | 0.91 | 0.71-1.17 |
| Educational level | Illiterate | 980 (5.8%) | 26,503 (6.1%) | 27,483 (6.1%) | - | 1 | Reference |
|  | Middle school first cycle | 4,142 (24.4%) | 115,642 (26.8%) | 119,784 (26.7%) | 0.388 | 0.97 | 0.90-1.04 |
|  | Middle school second cycle | 2,944 (17.3%) | 81,650 (18.9%) | 84,594 (18.8%) | 0.514 | 0.98 | 0.91-1.05 |
|  | High school | 5,344 (31.4% | 141,230 (32.7%) | 146,574 (32.6%) | 0.526 | 1.02 | 0.95-1.10 |
|  | University education | 3,278 (19.3%) | 62,581 (14.5%) | 65,859 (14.7%) | <0.001 | 1.42 | 1.32-1.52 |
|  | Does not apply | 307 (1.8%) | 4,583 (1.1%) | 4,890 (1.1%) | <0.001 | 1.81 | 1.59-2.07 |
| Region | Urban | 36,956 (95.8%) | 932,390 (94.5%) | 969,346 (94.5%) | <0.001 | 1 | Reference |
|  | Rural | 1,509 (3.9%) | 51,125 (5.2%) | 52,634 (5.1%) | <0.001 | 0.74 | 0.71-0.78 |
|  | Peri-urban | 120 (0.3%) | 3,166 (0.3% | 3,286 (0.3%) | 0.664 | 0.96 | 0.80-1.15 |
| Living in a Flu outbreak region | Yes | 4,670 (31.2%) | 74,576 (27.5%) | 79,246 (27.7%) | <0.001 | 1.20 | 1.16-1.24 |
|  | No | 10,281 (68.8%) | 197,043 (72.5%) | 207,324 (72.3%) | - | 1 | Reference |

We presented the number of patients and percentage. OR, odds ratio; 95% CI, 95% confidence interval.

We did the statistical analysis using the chi-square test. We used an alpha error of 0.05.

| **Supplementary Table 2.** Association between clinical signals and the asthma diagnosis in hospitalized patients with severe acute respiratory syndrome (SARS) due to coronavirus disease (COVID-19) in Brazil. | | | | | | | |
| --- | --- | --- | --- | --- | --- | --- | --- |
| **Clinical signs** | **Group** | **Asthma** | | **Total** | **P-value** | **OR** | **95% CI** |
|  |  | **Yes** | **No** |  |  |  |  |
| Fever | Yes | 24,978 (66.4%) | 616,056 (62.5%) | 641,034 (62.6%) | <0.001 | 1.19 | 1.16-1.22 |
|  | No | 12,633 (33.6%) | 370,271 (37.5%) | 382,904 (37.4%) | - | 1 | Reference |
| Cough | Yes | 31,765 (81.2%) | 758,720 (75.4%) | 790,485 (75.6%) | <0.001 | 1.67 | 1.63-1.71 |
|  | No | 7,371 (18.8%) | 247,395 (24.6%) | 254,766 (24.4%) | - | 1 | Reference |
| Sore throat | Yes | 7,777 (24.0%) | 197,156 (21.7%) | 204,933 (21.8%) | <0.001 | 1.14 | 1.11-1.17 |
|  | No | 24,574 (76.0%) | 712,504 (78.3%) | 737,078 (78.2%) |  | 1 | Reference |
| Dyspnea | Yes | 34,799 (86.4%) | 789,748 (78.4%) | 824,547 (78.7%) | <0.001 | 1.75 | 1.70-1.80 |
|  | No | 5,494 (13.6%) | 218,213 (21.6%) | 223,707 (21.3%) | - | 1 | Reference |
| Respiratory discomfort | Yes | 26,879 (72.9%) | 642,218 (66.2%) | 669,097 (66.4%) | <0.001 | 1.37 | 1.34-1.40 |
|  | No | 10,010 (27.1%) | 328,162 (33.8%) | 338,172 (33.6%) | - | 1 | Reference |
| SpO_2_ <95% | Yes | 29,812 (77.7%) | 728,998 (74.1%) | 758,810 (74.2%) | <0.001 | 1.22 | 1.19-1.25 |
|  | No | 8,533 (22.3%) | 254,864 (25.9%) | 263,397 (25.8%) | - | 1 | Reference |
| Diarrhea | Yes | 6,447 (19.9%) | 151,043 (16.7%) | 157,490 (16.8%) | <0.001 | 1.24 | 1.20-1.27 |
|  | No | 25,944 (80.1%) | 752,127 (83.3%) | 778,071 (83.2%) | - | 1 | Reference |
| Abdominal pain | Yes | 2,621 (10.0%) | 64,824 (8.0%) | 67,445 (8.1%) | <0.001 | 1.28 | 1.23-1.33 |
|  | No | 23,507 (90.0%) | 743,235 (92.0%) | 766,742 (91.9%) | - | 1 | Reference |
| Fatigue | Yes | 10,857 (39.2%) | 283,050 (34.0%) | 293,907 (34.1%) | <0.001 | 1.25 | 1.23-1.33 |
|  | No | 16,866 (60.8%) | 550,235 (66.0%) | 567,101 (65.9%) | - | 1 | Reference |
| Loss of smell | Yes | 4,226 (16.0%) | 109,175 (13.4%) | 113,401 (13.5%) | <0.001 | 1.23 | 1.18-1.27 |
|  | No | 22,231 (84.0%) | 703,268 (86.6%) | 725,499 (86.5%) | - | 1 | Reference |
| Loss of taste | Yes | 4,247 (16.1%) | 111,132 (13.7%) | 115,379 (13.8%) | <0.001 | 1.21 | 1.17-1.25 |
|  | No | 22,118 (83.9%) | 700,205 (86.3%) | 722,323 (86.2%) | - | 1 | Reference |
| Vomit | Yes | 3,932 (12.4%) | 92,081 (10.3%) | 96,013 (10.4%) | <0.001 | 1.23 | 1.19-1.28 |
|  | No | 27,802 (87.6%) | 802,664 (89.7%) | 830,466 (89.6%) | - | 1 | Reference |
| Other clinical signs | Yes | 15,175 (47.6%) | 357,724 (41.4%) | 372,899 (41.6%) | <0.001 | 1.29 | 1.26-1.32 |
|  | No | 16,690 (52.4%) | 506,406 (58.6%) | 523,096 (58.4%) | - | 1 | Reference |

We presented data as the number of patients and percentage. OR, odds ratio; 95% CI, 95% confidence interval; SpO_2_, oxygen saturation.

We did the statistical analysis using the chi-square test. We used an alpha error of 0.05.

| **Supplementary Table 3.** Association between comorbidities and the asthma diagnosis in hospitalized patients with severe acute respiratory syndrome (SARS) due to Coronavirus Disease (COVID-19) in Brazil. | | | | | | | |
| --- | --- | --- | --- | --- | --- | --- | --- |
| **Comorbidities** | **Group** | **Asthma** | | **Total** | **P-value** | **OR** | **95% CI** |
|  |  | **Yes** | **No** |  |  |  |  |
| Puerperal | Yes | 177 (0.6%) | 2,880 (0.3%) | 3,057 (0.3%) | <0.001 | 2.39 | 2.05-2.78 |
|  | No | 27,634 (99.4%) | 1,073,514 (99.7%) | 1,101,148 (99.7%) | - | 1 | Reference |
| Cardiopathy | Yes | 14,894 (45.4%) | 325,401 (30.0%) | 340,295 (30.5%) | <0.001 | 1.94 | 1.90-1.99 |
|  | No | 17,881 (54.6%) | 759,269 (70.0%) | 777,150 (69.5%) | - | 1 | Reference |
| Hematologic diseases | Yes | 689 (2.5%) | 6,573 (0.6%) | 7,262 (0.7%) | <0.001 | 4.12 | 3.81-4.46 |
|  | No | 27,383 (97.5%) | 1,076,517 (99.4%) | 1,103,900 (99.3%) | - | 1 | Reference |
| Down Syndrome | Yes | 391 (1.4%) | 3,015 (0.3%) | 3,406 (0.3%) | <0.001 | 5.07 | 4.56-5.63 |
|  | No | 27,662 (98.6%) | 1,080,518 (99.7%) | 1,108,180 (99.7%) | - | 1 | Reference |
| Hepatic diseases | Sim | 678 (2.4%) | 7,997 (0.7%) | 8,675 (0.8%) | <0.001 | 3.34 | 3.08-3.61 |
|  | No | 27,268 (97.6%) | 1,072,872 (99.3%) | 1,100,140 (99.2%) | - | 1 | Reference |
| Diabetes mellitus | Yes | 9,847 (31.7%) | 231,852 (21.4%) | 241,699 (21.7%) | <0.001 | 1.71 | 1.67-1.75 |
|  | No | 21,215 (68.3%) | 852,397 (78.6%) | 873,612 (78.3%) | - | 1 | Reference |
| Neurological diseases | Yes | 1,537 (5.4%) | 34,443 (3.2%) | 35,980 (3.2%) | <0.001 | 1.75 | 1.66-1.85 |
|  | No | 26,698 (94.6%) | 1047,846 (96.8%) | 1,074,544 (96.8%) | - | 1 | Reference |
| Immunosuppressive diseases | Yes | 1,046 (3.7%) | 22,738 (2.1%) | 23,784 (2.1%) | <0.001 | 1.80 | 1.69-1.92 |
|  | No | 26,980 (96.3%) | 1057,173 (97.9%) | 1,084,153 (97.9%) | - | 1 | Reference |
| Kidney diseases | Yes | 1,541 (5.5%) | 33,919 (3.1%) | 35,460 (3.2%) | <0.001 | 1.80 | 1.71-1.90 |
|  | No | 26,408 (94.5%) | 1046,225 (96.9%) | 1,072,633 (96.8%) | - | 1 | Reference |
| Obesity | Yes | 7,276 (24.6%) | 88,845 (8.3%) | 96,121 (8.7%) | <0.001 | 3.60 | 3.50-3.70 |
|  | No | 22,350 (75.4%) | 982,842 (91.7%) | 1,005,192 (91.3%) | - | 1 | Reference |
| Other comorbidities | Yes | 12,495 (41.6%) | 261,418 (24.9%) | 273,913 (25.3%) | <0.001 | 2.15 | 2.10-2.20 |
|  | No | 17,544 (58.4%) | 789,351 (75.1%) | 806,895 (74.7%) | - | 1 | Reference |

We presented data as the number of patients and percentage. OR, odds ratio; 95% CI, 95% confidence interval.

We did the statistical analysis using the chi-square test. We used an alpha error of 0.05.

| **Supplementary Table 4.** Association between flu vaccine, antiviral treatment, nosocomial infection, image findings from the thorax, follow-up during the hospitalization, outcome, and the asthma diagnosis in hospitalized patients with severe acute respiratory syndrome (SARS) due to coronavirus disease (COVID-19) in Brazil. | | | | | | | |
| --- | --- | --- | --- | --- | --- | --- | --- |
| **Patient’s characteristics** | **Group** | **Asthma** | | **Total** | **P-value** | **OR** | **95% CI** |
|  |  | **Yes** | **No** |  |  |  |  |
| Influenza Virus vaccine in the last campaign | Yes | 5,911 (30.5%) | 114,238 (22.1%) | 120,149 (22.4%) | <0.001 | 1.55 | 1.50-1.60 |
|  | No | 13,458 (69.5%) | 402,337 (77.9%) | 415,795 (77.6%) |  | 1 | Reference |
| Antiviral use to treat the clinical signs | Yes | 4,019 (12.4%) | 64,199 (8.0%) | 68,218 (8.2%) | <0.001 | 1.63 | 1.57-1.68 |
|  | No | 28,386 (87.6%) | 736,895 (92.0%) | 765,281 (91.8%) |  | 1 | Reference |
| Nosocomial infection | Yes | 634 (1.9%) | 17,888 (2.0%) | 18,522 (2.0%) | 0.049 | 0.92 | 0.85-0.99 |
|  | No | 33,134 (98.1%) | 862,788 (98.0%) | 895,922 (98.0%) |  | 1 | Reference |
| Thorax X-ray findings | Normal | 1,017 (4.1%) | 17,885 (3.0%) | 18,902 (3.0%) |  | 1 | Reference |
|  | Interstitial infiltrate | 6,320 (25.4%) | 143,680 (24.0%) | 150,000 (24.0%) | <0.001 | 0.77 | 0.72-0.83 |
|  | Consolidation | 911 (3.7%) | 16,123 (2.7%) | 17,034 (2.7%) | 0.910 | 0.99 | 0.91-1.09 |
|  | Mixed findings | 961 (3.9%) | 20,455 (3.4%) | 21,416 (3.4%) | <0.001 | 0.83 | 0.75-0.90 |
|  | Other | 3,165 (12.7%) | 59,186 (9.9%) | 62,351 (10.0%) | 0.101 | 0.94 | 0.87-1.01 |
|  | Not performed | 12,463 (50.2%) | 341,786 (57.0%) | 354,249 (56.8%) | - | - | - |
| Thorax computerized tomography findings | COVID-19 typical | 16,590 (66.2%) | 434,277 (65.5%) | 450,867 (65.5%) | <0.001 | 0.81 | 0.78-0.85 |
|  | COVID-19 undetermined | 800 (3.2%) | 16,141 (2.4%) | 16,941 (2.5%) | - | 1 | Reference |
|  | COVID-19 atypical | 407 (1.6%) | 8,613 (1.3%) | 9,020 (1.3%) | - | 1 | Reference |
|  | Negative for pneumonia | 138 (0.6%) | 1,618 (0.2%) | 1,756 (0.3%) | - | 1 | Reference |
|  | Other | 1,229 (4.9%) | 28,357 (4.3%) | 29,586 (4.3%) | - | 1 | Reference |
|  | Not performed | 5,883 (23.5%) | 173,852 (26.2%) | 179,735 (26.1%) | - | - | - |
| Closure criteria | Laboratory criterion | 38,764 (91.4%) | 961,737 (91.0%) | 1,000,501 (91.0%) |  | 1 | Reference |
|  | Clinical epidemiological findings | 387 (0.9%) | 10,693 (1.0%) | 11,080 (1.0%) | 0.040 | 0.90 | 0.81-0.99 |
|  | Clinical findings | 805 (1.9%) | 23,864 (2.3%) | 24,669 (2.2%) | <0.001 | 0.84 | 0.78-0.90 |
|  | Clinical and image findings | 2,444 (5.8%) | 60,614 (5.7%) | 63,058 (5.7%) | 0.995 | 1.00 | 0.96-1.04 |
| Need for intensive care unit | Yes | 14,928 (37.8%) | 351,422 (36.3%) | 366,350 (36.4%) | <0.001 | 1.07 | 1.05-1.09 |
|  | No | 24,513 (62.2%) | 616,468 (63.7%) | 640,981 (63.6%) |  | 1 | Reference |
| Need for mechanical ventilatory support | Invasive | 8,105 (20.8%) | 198,981 (20.8%) | 207,086 (20.8%) | 0.023 | 0.96 | 0.93-0.99 |
|  | Non-invasive | 23,428 (60.0%) | 581,039 (60.7%) | 604,467 (60.7%) | <0.001 | 0.95 | 0.93-0.98 |
|  | None required* | 7,502 (19.2%) | 177,438 (18.5%) | 184,940 (18.6%) |  | 1 | Reference |
| Evolution (Outcome) | Cure | 27,719 (69.7%) | 651,453 (65.8%) | 679,172 (66.0%) | <0.001 | 1 | Reference |
|  | Death | 12,053 (30.3%) | 338,443 (34.2%) | 350,496 (34.0%) |  | 0.84 | 0.82-0.86 |
| COVID-19 vaccine (vaccine against SARS-CoV-2) | Yes | 4,366 (34.9%) | 119,721 (27.9%) | 124,087 (28.1%) | <0.001 | 1.39 | 1.34-1.44 |
|  | No | 8,127 (65.1%) | 309,408 (72.1%) | 317,535 (71.9%) |  | 1 | Reference |

We presented data as the number of patients and percentage. OR, odds ratio; 95% CI, 95% confidence interval.

We did the statistical analysis using the chi-square test. We used an alpha error of 0.05.

^*^, or mechanical ventilation was not performed due to a lack of equipment.

| **Supplementary Table 5.** Multivariate analysis predicts the chance of death among hospitalized patients with severe acute respiratory syndrome (SARS) due to coronavirus disease (COVID-19) in Brazil. | | | | | | | | |
| --- | --- | --- | --- | --- | --- | --- | --- | --- |
| **Patient’s characteristics** | **B** | **SE** | **Wald** | **df** | **P-value** | **OR** | **95% CI** | |
|  |  |  |  |  |  |  | **Lower limit** | **Upper limit** |
| Sex (Female) | -0.273 | 0.014 | 398.311 | 1 | <0.001 | 0.761 | 0.741 | 0.782 |
| Age (y.o.) |  |  |  |  |  |  |  |  |
| 0-12 y.o. (Reference) |  |  | 6,523.285 | 5 | <0.001 |  |  |  |
| 13-24 y.o. | 1.061 | 0.194 | 29.763 | 1 | <0.001 | 2.888 | 1.973 | 4.228 |
| 25-60 y.o. | 1.577 | 0.180 | 76.856 | 1 | <0.001 | 4.842 | 3.403 | 6.889 |
| 61-72 y.o. | 2.352 | 0.180 | 170.928 | 1 | <0.001 | 10.509 | 7.386 | 14.952 |
| 73-85 y.o. | 2.863 | 0.180 | 253.160 | 1 | <0.001 | 17.508 | 12.305 | 24.910 |
| +85 y.o. | 3.357 | 0.181 | 344.563 | 1 | <0.001 | 28.704 | 20.138 | 40.916 |
| Race |  |  |  |  |  |  |  |  |
| White (Reference) |  |  | 463.214 | 4 | <0.001 |  |  |  |
| Black | 0.164 | 0.029 | 31.470 | 1 | <0.001 | 1.178 | 1.113 | 1.248 |
| Asian | 0.036 | 0.069 | .271 | 1 | 0.603 | 1.036 | 0.906 | 1.186 |
| *Pardos* (multiracial background) | 0.307 | 0.015 | 428.754 | 1 | <0.001 | 1.359 | 1.320 | 1.399 |
| Indigenous | 0.792 | 0.114 | 47.951 | 1 | <0.001 | 2.209 | 1.765 | 2.764 |
| Educational level |  |  |  |  |  |  |  |  |
| Illiterate (Reference) |  |  | 528.938 | 5 | <0.001 |  |  |  |
| Middle school first cycle | -0.139 | 0.027 | 27.212 | 1 | <0.001 | 0.871 | 0.826 | 0.917 |
| Middle school second cycle | -0.215 | 0.029 | 56.452 | 1 | <0.001 | 0.806 | 0.762 | 0.853 |
| High school | -0.373 | 0.028 | 172.948 | 1 | <0.001 | 0.689 | 0.651 | 0.728 |
| University education | -0.596 | 0.032 | 340.855 | 1 | <0.001 | 0.551 | 0.517 | 0.587 |
| Does not apply | 0.494 | 0.211 | 5.467 | 1 | 0.019 | 1.639 | 1.083 | 2.480 |
| Living in a Flu outbreak region | 0.169 | 0.016 | 116.722 | 1 | <0.001 | 1.184 | 1.148 | 1.221 |
| Puerperal | -0.300 | 0.139 | 4.677 | 1 | 0.031 | 0.741 | 0.564 | 0.972 |
| Comorbidities |  |  |  |  |  |  |  |  |
| Cardiopathies | 0.090 | 0.014 | 41.315 | 1 | <0.001 | 1.094 | 1.064 | 1.124 |
| Hematologic diseases | 0.260 | 0.063 | 17.208 | 1 | <0.001 | 1.297 | 1.147 | 1.467 |
| Hepatic diseases | 0.539 | 0.058 | 86.304 | 1 | <0.001 | 1.715 | 1.530 | 1.922 |
| Asthma | -0.238 | 0.037 | 41.437 | 1 | <0.001 | 0.788 | 0.733 | 0.847 |
| Diabetes mellitus | 0.172 | 0.014 | 144.444 | 1 | <0.001 | 1.187 | 1.155 | 1.221 |
| Neurological diseases | 0.474 | 0.030 | 248.534 | 1 | <0.001 | 1.607 | 1.515 | 1.704 |
| Immunosuppressive diseases | 0.642 | 0.033 | 368.670 | 1 | <0.001 | 1.900 | 1.779 | 2.029 |
| Kidney diseases | 0.641 | 0.029 | 480.927 | 1 | <0.001 | 1.899 | 1.793 | 2.011 |
| Obesity | 0.410 | 0.022 | 352.891 | 1 | <0.001 | 1.507 | 1.444 | 1.573 |
| Constant | -2.748 | 0.180 | 233.168 | 1 | <0.001 | 0.064 |  |  |
| ^a^. Patient’s characteristics included in step one: sex, age, race, educational level, comorbidities, place of residence, and living in a Flu outbreak region. In the analyses, we removed the Down Syndrome and the place of residence as predictors.  Df, degrees of freedom; SE, standard error; OR, odds ratio; 95% CI, 95% confidence interval.  R^2^ of Cox and Snell=0.129; R^2^ of Nagelkerke=0.176; Chi-square=15,051.669; df=27.  We used an alpha error of 0.05. | | | | | | | | |
